# Effect of Colorants and Process Parameters on the Properties of Dope-Dyed Polylactic Acid Multifilament Yarns

**DOI:** 10.3390/polym14225021

**Published:** 2022-11-19

**Authors:** Naveen Kumar Balakrishnan, Stefan Siebert, Christoph Richter, Robert Groten, Gunnar Seide

**Affiliations:** 1 Aachen-Maastricht Institute for Biobased Materials (AMIBM), Maastricht University, Brightlands Chemelot Campus, Urmonderbaan 22, 6167 RD Geleen, The Netherlands; 2 Department of Textile and Clothing Technology, Niederrhein University of Applied Sciences, Campus Moenchengladbach, Webschulstrasse 31, 41065 Moenchengladbach, Germany

**Keywords:** fiber spinning, dope dyeing, nucleating agent, crystallinity, sustainability, biobased polymer, biopolymer processing, biobased textiles

## Abstract

The color of textile fibers is typically imparted by submersion in a high-temperature dye bath. However, the treatment of the effluent is challenging and the textile industry is therefore a major source of water pollution. Current fashion trends favor biobased polymers such as polylactic acid (PLA) but exhaust dyeing at high temperatures causes hydrolytic degradation, reducing the crystallinity and tenacity of the yarn. To preserve the mechanical properties of PLA-based textiles, an alternative to exhaust dyeing called dope dyeing can be used, wherein colorants are incorporated into the polymer matrix during melt spinning. We evaluated this process by dope dyeing PLA with several colorants, then testing the thermal, physical, and mechanical properties of the yarn and the physical properties of circular-knitted fabrics. Although the colorants affected the crystallization behavior at lower cooling rates, during the melt-spinning process, the drawing speed had a greater effect on the crystallinity and mechanical properties of the dyed yarn. Scanning electron microscopy revealed that the colorants were well dispersed in the PLA matrix. We found that the colorants did not affect the physical properties of the knitted fabric. Our results can be used to develop more environmentally beneficial dope-dyed PLA yarn with improved mechanical properties.

## 1. Introduction

Synthetic fiber production has doubled in the last two decades, reaching 80.9 million tonnes in 2020, and the most common material is polyethylene terephthalate (PET), accounting for 48 million tonnes [1]. Because PET is an oil-based polymer, its production from nonrenewable raw material, and its ultimate disposal contribute to climate change. The textile industry is therefore shifting towards more sustainable and biobased and/or biodegradable polymers, which reached a production volume of 2.11 million tonnes in 2020, with polylactic acid (PLA) accounting for 18.7% of this total [2]. PLA can be produced from renewable resources such as corn, sugar beet, and wheat, and it currently accounts for less than 0.03% of global corn production, thus representing negligible competition with food crops. Although often described as biodegradable, PLA is only compostable under industrial conditions [3]. The production of 1 tonne of PLA consumes 42 GJ of energy and releases 1.3 tonnes of CO_2_, ~40% less than PET [4]. Furthermore, PLA fibers are characterized by inherently better moisture management, a higher limiting oxygen index (LOI), and enhanced wicking properties compared to PET fibers, making them suitable for use in sustainable clothing [5].

Despite the advantages of PLA, its commercial production is limited by its thermolability and low heat resistance due to its glass transition temperature (T_g_) of ~55 °C [6]. The heat resistance of PLA can be improved by increasing its crystallinity, but the inherent low crystallization rate of the polymer leads to products with low crystallinity, which hinders conventional exhaust dyeing [7,8]. PET is exhaust dyed at 130 °C without loss of integrity, but PLA is hydrolyzed under these conditions, weakening the fibers [9]. However, dyeing at lower temperatures results in poor dye take-up compared to PET [3,10,11]. The exhaust dyeing of PLA with indigoid compounds is possible at 110 °C [11,12]. Furthermore, PLA fibers modified with polyhedral oligomeric silsesquioxane nanoparticles also showed better dyeability than pure PLA [13]. The dyeing of recycled PLA fibers using selected disperse dyes achieved satisfactory exhaustion rates at 80 °C, but the light fastness of the fibers was poor [10]. Natural dye from flowers has also been used to produce yellow PLA films [14]. The tensile strength of PLA declines with increasing dyeing time, probably reflecting the impact on fiber crystallinity [6,15].

Despite its widespread use, conventional exhaust dyeing is unsustainable because toxic chemicals are needed to fix colorants to the polymer and on top of it, 10–25% of the dye is lost during the process, 2–20% of which is directly discharged into rivers and streams along with the toxic fixatives [16]. The textile industry is therefore the second greatest global polluter of water resources [17]. Exhaust dying at high temperatures also consumes energy, and the high temperatures not only cause the degradation of labile fibers such as PLA but also affect crystallinity and lead to shrinkage, disrupting the fiber orientation [6]. In comparison, mass coloration or dope dyeing involves the incorporation of colorants into the polymer melt during fiber production [18]. The dope dyeing of PLA with carbon black was shown to improve the mechanical properties of the fibers [19]. A comparison of dope dyeing and exhaust dyeing of polyamide 6 revealed that dope dyeing reduces water use by ~40%, chemical use by 97%, and waste by ~50% and also reduces the overall number of processing steps [20].

Although dope dyeing has many advantages, only 5% of polyester fibers are dope dyed, and the proportion is even lower for PLA [18]. Like PLA, polypropylene (PP) is also characterized by slow crystallization, but this has been controlled and optimized by using colorants as nucleating agents [21]. We recently reported a similar nucleating effect when using selected pigments in PLA [22]. However, the effect of colorants on the mechanical, rheological, and thermal properties of PLA during dope dyeing has not been studied in detail. Herein, we describe the dope dyeing of PLA with fossil-based and biobased colorants. We compared the biobased pigment pink PR122 and the potential biobased colorant alizarin to the fossil-based pigments green 7, blue 15:1, and yellow 155. We incorporated different weight percentages of the colorants into PLA melts and determined their effect on the rheological and thermal properties of yarn, including their propensity for degradation. We also used different draw ratios to investigate the influence of additives and process parameters on the mechanical properties and crystallinity of the PLA yarns. Finally, we prepared circular-knitted fabrics from the melt-spun yarns and investigated their air permeability, water vapor permeability, and Martindale abrasion resistance. We compared the performance of the different colorants to assess the potential of biobased colorants for the development of environmentally beneficial dope-dyed PLA yarns with improved properties.

## 2. Materials and Methods

### 2.1. Materials

PLA grade L130 (TotalEnergies-Corbion, Gorinchem, Netherlands) was used for all experiments and has the following manufacturer-specified properties: L-content ≥ 99%, T_g_ ~60 °C, and melt flow index = 24 g/10 min at 210 °C/2.16 kg. We purchased alizarin (Sigma-Aldrich, St Louis, MO, USA), whereas the pigments blue 15:1, green 7, pink PR122, and pigment yellow 155 were kindly donated by Clariant (Muttenz, Switzerland). The chemical structure of the colorants are presented below in Table 1.

### 2.2. Compounding

We dried PLA and the colorants under a vacuum at 80 °C overnight to reduce the moisture content. The materials were compounded using a KETSE 20/40 twin-screw extruder (Brabender, Duisburg, Germany) with a screw diameter (D) of 20 mm and a length of 40D. Master batches containing 5% (*w*/*w*) of each colorant were prepared (named A5, B5, G5, P5, and Y5). The extrudates were cooled in a water bath before granulation.

### 2.3. Melt Spinning

**Figure 1:**
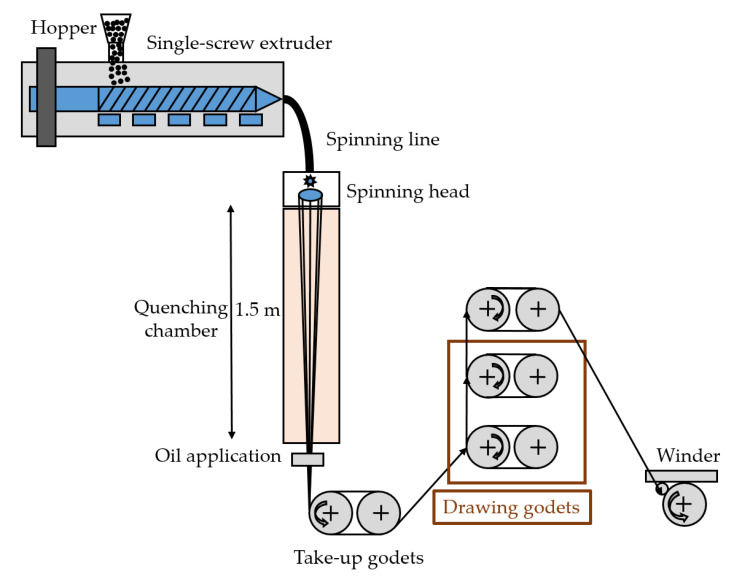
Schematic illustration of the pilot-scale melt-spinning device.

Fibers were prepared using an FET-100 series pilot-scale melt-spinning machine (Fibre Extrusion Technology, Leeds, UK) featuring a spinneret with 48 holes (diameter 0.25 mm) and an L/D ratio of 2 (Figure 1). The materials were dried again at 80 °C overnight under vacuum before spinning at a temperature of 200 °C. We maintained a constant extruder pressure of 60 bars and a constant throughput of 45 g/min at a winding speed of 1200 m/min, but we applied three different draw ratios.

The master-batch granules were diluted with pure PLA to achieve weight-percentages of 0.1%, 0.2%, and 0.3% for melt spinning. The first yarn was drawn fully from the melt, and no solid-state drawing (SSD) was applied. The second yarn was semi-drawn from the melt (medium SSD). The third yarn was only slightly drawn from the melt and was primarily drawn by SSD. We compared yarns produced with the highest weight percentage of colorants (0.3%) because, if no significant effects were observed at this concentration, we could rule out effects at lower concentrations without testing. The yarns were named using the convention C0x.y, wherein C is the first letter of the colorant name, x is the weight percentage, and y is the SSD ratio. For example, PLA.1 refers to the pure PLA yarn drawn with an SSD ratio of 1, and A03.2 refers to the yarn dyed with 0.3% (*w*/*w*) alizarin and drawn with an SSD ratio of 2. The complete set of yarns is presented in Table 2.

### 2.4. Knitting

The melt-spun yarn was knitted using a TK-83 circular knitting machine (Harry Lucas, Neumünster, Germany). A knitted fabric with a single jersey structure was produced on gauge E24/gg using 264 needles with a cylinder diameter of 3½ inches. A knitted fabric was prepared from PLA and dyed PLA yarns produced at an SSD ratio of 1 and a colorant content of 0.3% (*w*/*w*). All fabrics were prepared using the same knitting parameters (stitch, take-off, and knitting speed).

### 2.5. Characterization of Colorants

Differential scanning calorimetry (DSC) was used to reveal any thermal transitions during processing. We applied one heating and one cooling cycle from 25 °C and 250 °C at a heating rate of 10 °C/min using a SC 214 device (Netzsch, Selb, Germany). The morphology of the colorants was determined by scanning electron microscopy (SEM) using a JSM-IT200 device (Jeol, Freising, Germany). The images were acquired in secondary electron mode with an acceleration tension of 15 kV and are presented at 1000× magnification.

### 2.6. Characterization of Master Batches

The masterbatches of PLA with 5% (*w*/*w*) of colorants were analyzed by DSC from 25 °C to 200 °C to investigate the effect of colorants on thermal transition temperatures. We applied two heating cycles and one cooling cycle. We used the second heating cycle to determine the effect of colorants on any change in thermal transition, and we used the first cooling cycle to investigate any change in crystallization temperature (Tc). The T_g_, melting point (T_m_) and cold crystallization temperature (T_cc_) were also compared.

Rheological analysis was carried out using a Discovery Hybrid Rheometer (DHR1) from TA Instruments (New Castle, DE, USA). Angular frequencies were applied over the range 1 to 624 rad/s using a 25 mm plate plate setup to investigate the effect of colorants on the viscosity of PLA, and complex viscosity values were compared at the same angular frequency (10 rad/s).

Fourier-transform infrared (FTIR) spectroscopy was used to investigate interactions between PLA and the colorants. FTIR spectra were recorded on a PerkinElmer 400 FT(N)IR device (Waltham, MA, USA) in transmission mode by completing 64 scans between 4000 and 500/cm with a resolution of 2/cm.

The cross-section of the master batches was analyzed by SEM as described above to determine the distribution of colorants. The samples were frozen in liquid nitrogen, fractured, and sputtered with gold prior to the measurements.

The average molecular weight (Mn), weight average molecular weight (Mw), and polydispersity index (PDI) of PLA and its master batches were determined by gel permeation chromatography (GPC) using a 1260 Infinity device (Agilent Technologies, Santa Clara, CA, USA). The mobile phase was hexafluoro-2-isopropanol (HFIP) containing 0.19% sodium trifluoroacetate. We prepared filtered solutions containing 5 mg samples and injected them into a 7 µm modified silica column. We used a standard polymethyl methacrylate polymer (1.0 × 10^5^ g/mol) for calibration.

### 2.7. Characterization of Yarn

DSC over the range 35 to 200 °C was used to determine the effect of colorants, their weight percentage, and the drawing parameters on the thermal transitions of PLA yarn. We measured the T_g_, T_m_, and T_cc_ and the degree of crystallinity (X_c_) of pure PLA and dyed PLA yarns. The melting enthalpy of 100% crystalline PLA is 93.7 J/g [28,29,30].

The yarn linear density (weight per length) was measured according to DIN EN ISO 1973, and mechanical properties (tenacity and elongation) were measured using a ZwickLine Z2.5 instrument (Zwick Roell, Ulm, Germany) according to DIN EN ISO 2062. A starting length (L_0_) of 200 mm was used, and the test was carried out at 200 mm/min. All tests were carried out in triplicate.

Commission Internationale de l’Eclairage (CIE) L*a*b* color coordinates were measured using a PCE CSM 7 colorimeter (PCE Instruments, Palm Beach, FL, USA) according to EN ISO 11664-4. An aperture size of 4 mm was used. L* is the lightness index and can vary from 0 (black) to 100 (white); a higher negative a* value indicates a stronger green color; a higher positive a* value indicates a stronger red color. Similarly, a higher negative b* value indicates a stronger blue color, and a higher positive b* value indicates a stronger yellow color.

The ultraviolet (UV) light stability of the colorants in dyed yarns was measured using a blue wool scale (BWS) test according to ISO 105-B02. We exposed the samples to UVA light (0.68 W/m^2^) at 340 nm and 50 °C. We prepared sample swatches from the yarn dyed with 0.3% (*w*/*w*) of each colorant, and the CIE L*a*b* coordinates were measured on the left, middle, and right. The middle part of the sample was always covered to prevent UV exposure. Samples showing a visual change at level 4 of the grayscale were given a preliminary score. The left part was then covered. The test was continued, and a final score was given when the right part matched level 3 of the grayscale. After 130 h, we evaluated the effect of the UV on the samples. At this point, we graded light-fastness against the BWS and assigned a score between 1 (very poor) and 8 (excellent).

### 2.8. Characterization of Knitted Fabric

The effect of the colorants on the air permeability and water vapor permeability of knitted fabrics was investigated using an FX 3300 tester (Textest Instruments, Schwerzenbach, Switzerland) and Permetest (Sensora, Liberec, Czech Republic). Air permeability was measured on all knitted fabrics with an air pressure of 200 Pa and a test area of 20 cm^2^ according to DIN EN ISO 9237. Water vapor permeability was measured according to DIN EN ISO 11092. Abrasion resistance was measured using an AquAbrasion 1819 Martindale abrasion test device (James Heal, Sterling, VA, USA). The test was carried out according to DIN EN ISO 12947-2 with 15,000 cycles. After 1000, 2000, 3000, 4000, 5000, 7500, and 10,000 cycles, the test was stopped, and images were captured to record the abrasion of the knitted fabric. The CIE L*a*b* color coordinates of the fabric were also measured, according to the procedure mentioned above, before, and after the Martindale test to determine the abrasion resistance of the colorants. Five samples of each knitted fabric were tested at a nominal pressure of 12 kPa.

## 3. Results and Discussion

### 3.1. Analysis of the Colorants

#### 3.1.1. Thermal Characteristics

**Figure 2:**
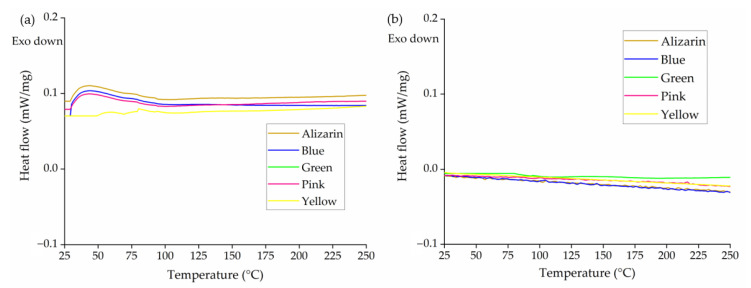
DSC thermograms of five colorants during (**a**) the heating cycle and (**b**) the cooling cycle.

Most colorants showed little to no thermal transition under our experimental conditions, as anticipated because their Tm lay beyond the tested temperature range (Figure 2). Even so, we observed a broad endothermic peak between 50 and 100 °C for the blue 15:1 and green 7 colorants and alizarin. Given that the two inorganic colorants were not dried before DSC and are based on copper complexes, this could indicate the evaporation of residual water. Similarly, the peak observed in the alizarin thermogram was assumed to represent water evaporation because the colorant is not expected to undergo other thermal transitions within this temperature range. Similar observations have been reported for TiO_2_ [31]. No significant peak was observed for any of the colorants during the cooling cycle. We used a temperature of 200 °C for the melt spinning of PLA, and no thermal transition was observed for any of the colorants below 200 °C, so no change in the physical state of the colorants was anticipated during melt spinning.

#### 3.1.2. Morphological Characteristics

**Figure 3:**
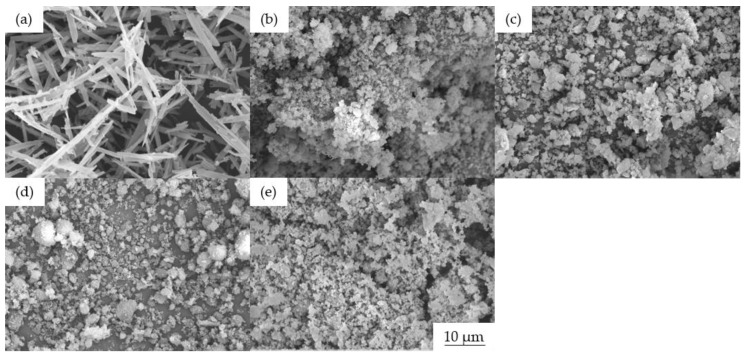
SEM images of the colorants (**a**) alizarin, (**b**) pigment blue 15:1, (**c**) pigment green 7, (**d**) pigment pink PR122, and (**e**) pigment yellow 155.

The colorants are supplied as tiny crystals in the shape of bricks, rods, or plates, but this structure can break down during processing due to the high temperature and/or shear forces, and the crystals can also dissolve in the surrounding medium [32]. SEM images revealed that alizarin has a needle-like structure, whereas the blue 15:1, green 7, and yellow 155 pigments have a plate-like structure, and pigment pink PR122 has a spherical morphology (Figure 3). All five colorants formed agglomerates, ranging in size from ~2 µm (blue 15:1, green 7 and yellow 155) to 2–5 µm (pink PR122) and 10–20 µm (alizarin).

### 3.2. Analysis of PLA Master Batches

#### 3.2.1. Thermal Characteristics

**Figure 4:**
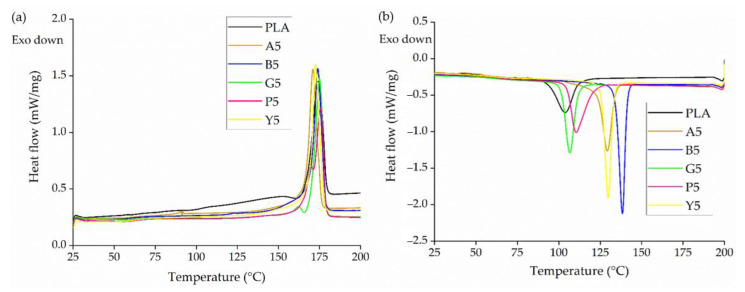
DSC thermograms of PLA and its master batches containing 5% (*w*/*w*) of each colorant during (**a**) the heating cycle and (**b**) the cooling cycle.

The DSC thermograms of PLA and its master batches containing 5% (*w*/*w*) of each colorant (A5, B5, G5, P5, and Y5) are presented in Figure 4. PLA and all five master batches showed a major melting peak at ~175 °C. However, master batches G5 and P5 also showed a second, minor melting peak at ~165 °C, which may indicate a defective crystal structure. The defective crystals melt and re-crystallize, then melt again at 175 °C [33]. During the cooling cycle, the T_c_ remained constant at ~100 °C for PLA as well as master batches G5 and P5, but the Tc of A5, B5, and Y5 was higher, with B5 showing the highest T_c_ of ~140 °C. These data indicate that these colorants have a nucleating effect on PLA and thus promote crystallization. Similar nucleating effects have been observed in the past for compounds containing small amounts of pigment in a PP matrix and by talc and bis(hydroxyethyl)terephthalate in the case of PLA [34,35].

#### 3.2.2. Rheological Characteristics

**Figure 5:**
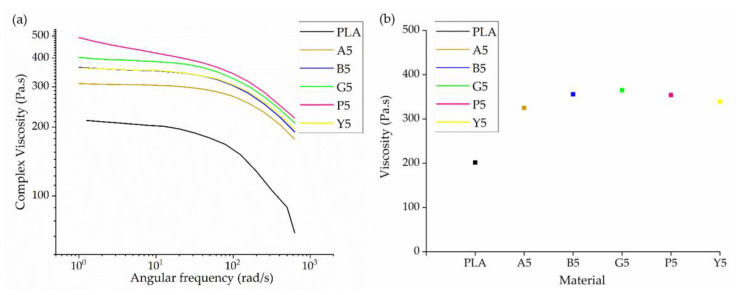
Rheological analysis of PLA and its master batches containing 5% (*w*/*w*) of each colorant. (**a**) Rheogram of PLA and the five master batches. (**b**) Complex viscosity of PLA and the five master batches at an angular frequency of 10/s.

The rheograms of PLA and its master batches containing 5% (*w*/*w*) of each colorant are presented in Figure 5. All six materials showed non-Newtonian shear thinning behavior, in which the viscosity declines with increasing shear rate. This behavior is typical of polymers at high temperatures [36,37,38]. To facilitate a visual comparison, the complex viscosity of PLA and its master batches are shown at an angular frequency of 10/s in Figure 5b.

The complex viscosity of PLA at an angular frequency of 10 rad/s was 201 Pa.s, but the value increased in the presence of each colorant. The highest increase of 47% was observed for master batch G5. Given that the colorants do not melt below 200 °C, they must act as anchors for the PLA chains, leading to an increase in melt viscosity. Furthermore, the polar groups in the colorants can interact with the polar groups in the PLA chain, hindering chain slippage and increasing the viscosity even further, as previously reported [22]. A similar increase in viscosity was reported when PLA was grafted with carbon black [39]. However, given the much lower weight percentage of colorants used for melt spinning (0.1–0.3%), we did not expect a substantial increase in viscosity or any effect on the behavior of the polymer.

#### 3.2.3. Molecular Interactions between the Colorants and PLA

**Figure 6:**
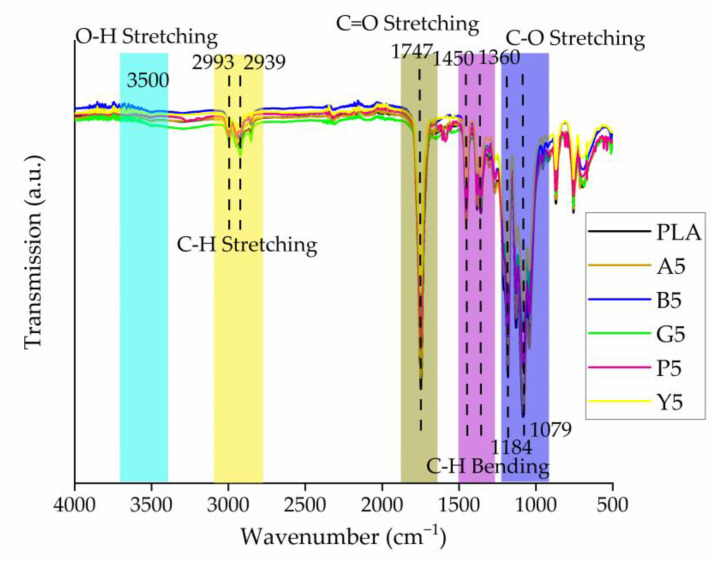
FTIR spectra of PLA and its master batches containing 5% (*w*/*w*) of each colorant.

The FTIR spectra of PLA and its master batches were very similar (Figure 6). They all featured the characteristic C-H stretching peaks at 2993/cm and 2939/cm and C-O bending at 1184/cm. However, the peak at 1747/cm, representing the C=O stretching of PLA, shifted to 1750/cm in all of the master batches. This may signify an interaction between the hydroxyl groups of PLA and the functional groups of colorants, which could promote the distribution of colorant molecules in the PLA matrix. A similar shift in the C=O stretching peak of PLA was also observed when PLA was mixed with Kenaf fibers, indicating the possibility of hydrogen bonding between the components [40]. The hydrogen bonding observed in our FTIR spectra may explain the increase in the viscosity of the PLA master batches. In the case of the P5 master batch, we observe additional peaks between wavenumbers of 1650/cm and 1580/cm. This can be attributed to the bending vibration of the amine (N-H) group present in the pigment [41,42].

#### 3.2.4. Morphological Analysis

**Figure 7:**
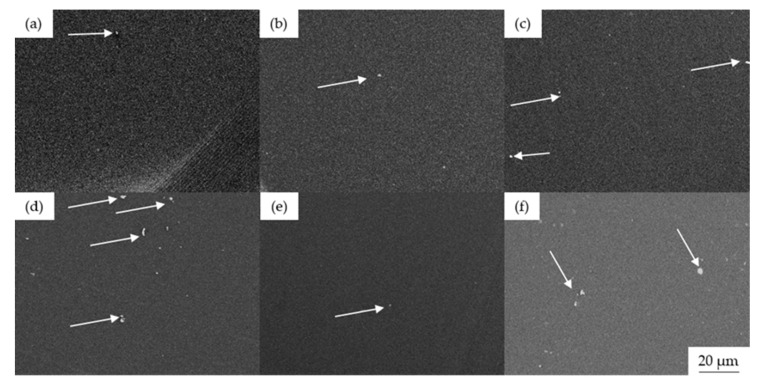
SEM images of cross-sectioned PLA and master batch granules: (**a**) pure PLA, (**b**) A5, (**c**) B5, (**d**) G5, (**e**) P5, and (**f**) Y5. Arrows indicate the position of aggregates.

The dispersion of colorants in the PLA matrix was investigated by SEM. Cross-section images of PLA (after the compounding simulation step) and the master batches revealed that three colorants (alizarin, pigment blue 15:1, and pink PR122) were well dispersed (Figure 7). We only observed a few small aggregates (~2 µm) in these samples, including the pure PLA, suggesting that they represented additives introduced by the manufacturer. However, we find more agglomerates in case of the master batches made with green 7 and yellow 155. In Section 3.1.2, we observed that these two pigments have plate-like structure, and this leads to the hypothesis that these structures are harder to disperse in PLA. Still, this contrasts with our previous study, in which we observed more abundant aggregates for almost all colorants [22]. However, in the previous study, a glass syringe with a plunger was used for spinning. The compounds were filled and melted inside the syringe barrel, and no shear was applied to break down the aggregates. In contrast, we applied shear during the preparation of our master batches and melt spinning to improve the mixing of the colorants. Furthermore, the colorants used here (and PLA) dissolve in solvents such as chloroform and dimethyl formamide, suggesting the Hansen solubility parameter of the colorants and PLA matrix are similar. FTIR spectroscopy provided evidence for the formation of hydrogen bonds, which also supports the compatibility of the colorants with PLA [43].

#### 3.2.5. GPC Analysis

**Figure 8:**
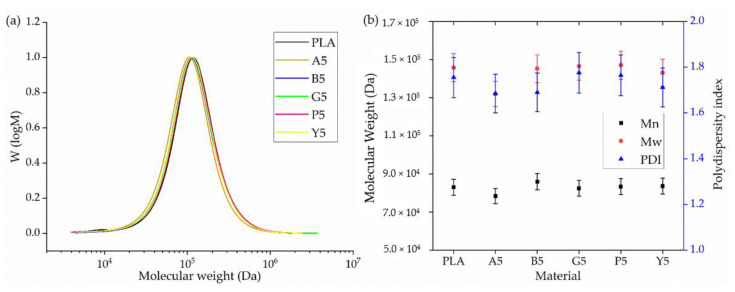
GPC analysis of PLA and its master batches containing 5% (*w*/*w*) of each colorant. (**a**) GPC elugram with normalized intensity on the y-axis. (**b**) Mean M_w_, M_n_, and PDI values ± SE (*n* = 3).

GPC analysis revealed no significant difference in the molecular weight or PDI of PLA and its master batches (Figure 8). The relative M_w_ of PLA was 145,690 Da; the M_n_ was 83,016 Da; and the PDI was 1.76. A5 had the lowest M_w_ (131,950 Da) and M_n_ (78,316 Da), whereas P5 had the highest M_w_ (147,060 Da) and M_n_ (83,355 Da). The PDI of all samples was ~1.70. These data confirmed that the addition of colorants did not lead to the degradation of PLA and that dope dyeing therefore avoids the polymer chain degradation that is typically observed during conventional exhaust dyeing.

### 3.3. Characterization of Yarns

#### 3.3.1. Mechanical Properties

**Figure 9:**
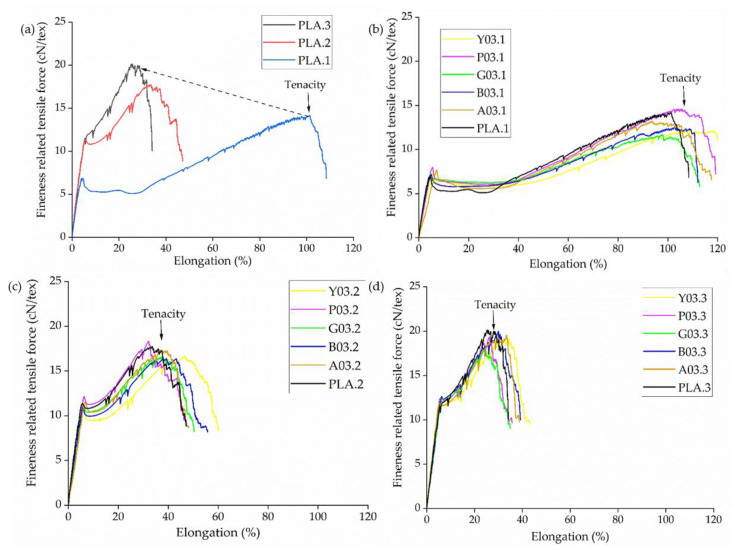
Tensilegrams of PLA and dyed yarns containing 0.3% (*w*/*w*) of each colorant at different SSD ratios. (**a**) Mean values of PLA yarns drawn at different SSD ratios. (**b**–**d**) Mean values of PLA and dyed yarns containing 0.3% (*w*/*w*) of each colorant drawn at SSD ratios of (**b**) 1, (**c**) 2, and (**d**) 3. Data are means of *n* = 5 samples.

The linear density of all yarns was measured to be 300 ± 5 dtex and was therefore considered 300 dtex for the tests. The tensile force vs. elongation graphs of PLA were compared at different SSD ratios (Figure 9a). We also compared PLA and dyed yarns containing 0.3% (*w*/*w*) of each colorant at SSD ratios of 1, 2, and 3 (Figure 9b–d).

The tenacity (tensile strength) of PLA yarns increased at higher SSD ratios (Figure 9a). The tenacity increased by almost 50% (from 14 to 20 cN/tex) when the SSD ratio increased from 1 to 3. At the same time, the elongation declined by 72% (from 94% to 26%). Increasing the SSD ratio was previously shown to increase tenacity and reduce elongation by stretching and orienting the polymer chains, leading to strain-induced crystallization [44,45]. The mechanical properties obtained from melt spun PLA yarn are similar to the values obtained by researchers in the past, when a maximum tenacity of about 30 cN/tex was reported [46,47]. There was no significant change in the mechanical properties of the yarn in the presence of colorants (Table 3). For example, at an SSD ratio of 3, pure PLA yarns showed the highest tenacity (20.03 cN/tex) and Y03.3 yarns the lowest (18.30 cN/tex), whereas A03.3 yarns showed the greatest elongation (31%) and Y03.3 yarns the lowest (25%). The properties of all of the other yarns produced also fall in the same range. This is a significant advantage compared to conventional exhaust dyeing, in which the higher temperature and prolonged dyeing time reduce the mechanical integrity of PLA fabrics [6,48].

#### 3.3.2. Physical Properties

**Figure 10:**
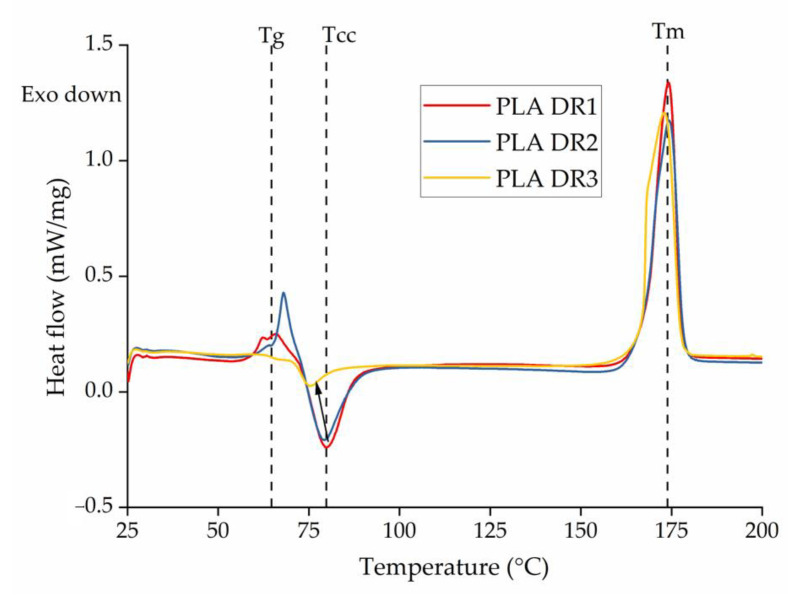
DSC thermogram of PLA yarns drawn at different ratios.

Increasing the SSD ratio did not change the T_m_ of the yarns (Figure 10). However, although the T_g_ remained constant at SSD ratios of 1 and 2, no T_g_ peak was observed at an SSD ratio of 3 (Figure 10). The tenacity of the yarns increased by 20% when the SSD ratio increased from 1 to 2, but the increase in crystallinity was negligible (Table 3). This suggests that drawing during spinning was sufficient to orient the polymer chains but insufficient to induce crystallization. However, at an SSD ratio of 3, the increase in tenacity was accompanied by a >1.5-fold increase in the degree of crystallinity, reaching 51.7% for the pure PLA fibers. Such an increase in drawing leading to increased crystallinity, to a maximum of 67%, was also reported by Mai et al. [44,49] and was also anticipated because higher SSD ratios orient the polymer chains, creating a more ordered structure that induces crystallization during processing. We also observed a shoulder in the melting peak for PLA fibers at an SSD ratio of 3, probably reflecting the formation of imperfect α′ crystals during drawing, which melt at a temperature lower than α crystals. Similar melting behavior has previously been observed for drawn PLA chains [44,49].

The higher crystallinity at an SSD ratio of 3 was also accompanied by a lower T_cc_. Cold crystallization occurs due to rapid quenching, when amorphous polymer chains that cannot crystallize during spinning relax and crystallize to form a more ordered structure. Two hypotheses could explain the lower Tcc. First, the crystallinity of the yarns may be higher in PLA.3, limiting cold crystallization and therefore reducing the peak crystallization temperature. Second, the polymer chains are already oriented in yarns drawn with higher SSD ratios, so less energy is needed for crystallization, and cold crystallization is therefore accelerated. If the first hypothesis were correct, the onset of cold crystallization would remain constant regardless of the SSD ratio. We therefore tested for the onset of crystallization and found that the temperature declined from 75.7 °C (PLA.1) to 72.1 °C (PLA.2) and 70.8 °C (PLA.3) as the SSD ratio increased. Because the onset of crystallization also changes, the first hypothesis is rejected and the second hypothesis becomes more plausible. This leads to the conclusion that, among the parameters considered, the orientation resulting from SSD is higher than that resulting from melt drawing.

**Figure 11:**
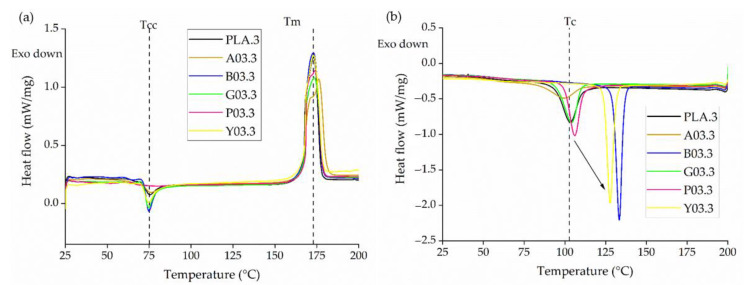
DSC thermogram of PLA and dyed yarns containing 0.3% (*w*/*w*) of each colorant at an SSD ratio of 3 during (**a**) the heating cycle and (**b**) the cooling cycle.

**Figure A1:**
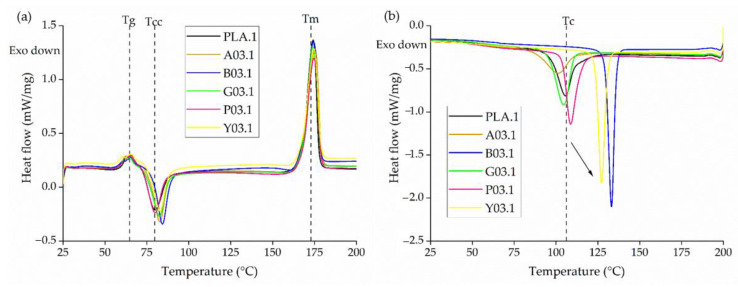
DSC thermograms of PLA and dyed yarns containing 0.3% (*w*/*w*) of each colorant at an SSD ratio of 1 during (**a**) the heating cycle and (**b**) the cooling cycle.

**Figure A2:**
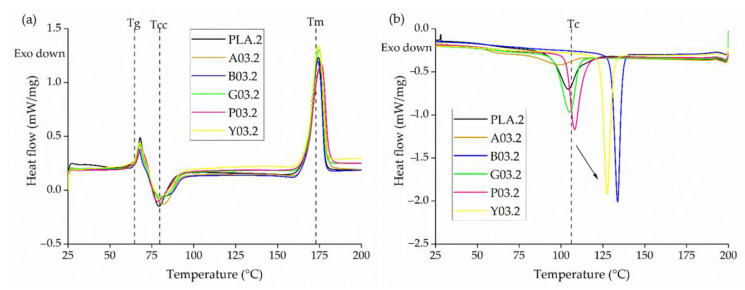
DSC thermograms of PLA and dyed yarns containing 0.3% (*w*/*w*) of each colorant at an SSD ratio of 2 during (**a**) the heating cycle and (**b**) the cooling cycle.

The DSC thermograms of pure PLA yarns and dyed yarns containing 0.3% (*w*/*w*) of each colorant produced at different SSD ratios are presented in Figure 11, and the corresponding T_g_, T_m_, T_cc_, T_c_ and X_c_ values are summarized in Table 4. The DSC curves for dyed yarn containing 0.1% and 0.2% (*w*/*w*) of each colorant were very similar and are provided in the appendix (Figure A1 and Figure A2). As described above for the master batches (Section 3.2.1), the Tc increased in the presence of 0.3% (*w*/*w*) of the blue 15:1 pigment and yellow 155 pigments because they act as strong nucleating agents. The T_c_ remained constant at 133 °C for the blue 15:1 pigment and 127 °C for the yellow 155 pigment, regardless of the weight percentage. Interestingly, although alizarin acted as a nucleating agent in master batch A5, the effect was not observed in the yarns, indicating that the nucleation effect only occurs at >0.3% (*w*/*w*) alizarin. 

Table 3 also shows that the T_g_, T_m_, T_cc_, and X_c_ values were unaffected by the presence of most colorants, but green 7 was an exception, with slightly lower values at an SSD of 3. However, the mechanical properties of the yarn were unchanged. This is probably because the colorant affects the crystallization process but does not interfere with polymer chain orientation. The degree of crystallinity of the yarns dyed with all other colorants drawn at an SSD ratio of 3 was 48–50%. This seems to be the case even for the blue 15:1 pigment and yellow 155 pigment showing nucleating effects. This is perhaps because DSC was carried out at lower cooling speeds of 10 °C/min, whereas higher cooling rates of >200 °C/s were applied during melt spinning. This suggests that, although some colorants act as strong nucleating agents at lower cooling rates, they do not have sufficient time to influence the crystallinity of the yarn during spinning. This differs from conventional dyeing, wherein changes in crystallinity are observed depending on the dyeing protocol. Only the green 7 colorant affected yarn crystallinity, but the effect was marginal and not enough to reduce mechanical strength. Dope dyeing is therefore advantageous because it offers better control over the crystallinity of the yarn.

#### 3.3.3. Color and UV Stability

The CIE L*a*b color values and BWS of the PLA and dyed yarns revealed that increasing the weight percentage of colorant resulted in a darker shade of yarn (Table 5). This was indicated either by the L value (whiteness index), for example, in yarns containing alizarin, or by both the L and a values in the case of the green 7 colorant, because lower L values indicate darker colors and negative a values represent greener hues. All five colorants were stable on exposure to UV light, scoring ≥ 7 in the BWS test. Although the color obtained by the exhaust dyeing of PLA with natural colorants like alizarin and indigo was good, the light fastness of PLA exhaust dyed with alizarin was reported to be low in the past even when PLA was modified with nanoparticles, whereas the dope dyeing approach used herein led to improved light fastness [13,50,51].

### 3.4. Characterization of Knitted Fabrics

#### 3.4.1. Water Vapor Permeability

Fabrics with greater water vapor permeability are considered to be more comfortable for daily use because they allow perspiration to evaporate [52]. The stitch density was kept constant in the knitting tests to avoid this parameter affecting the water vapor permeability [53]. We found that water vapor permeability was unaffected by the presence of colorants (Table 6). This agrees with previous studies of pigments added to silicone resin-based paints [54]. Water vapor permeability is affected by changes in the chemical structure, morphology, and surface characteristics of the fabric, which often occur during conventional dyeing because dye molecules needed to be fixed to the surface of fibers, thus ensuring color fastness. For example, a significant difference in water vapor permeability was reported, following the dyeing of cotton [55]. In contrast, dope dyeing involves the mixing of colorants with the polymer melt, causing the colorant to be distributed throughout the polymer matrix [18]. The absence of surface modification means that the water vapor permeability of knitted fabrics made from dyed PLA yarns is almost identical to that of pure PLA. It is also observed that the water vapor permeability of PLA fabric produced here is better than that of PET (reported to be 40%) and other PLA yarn (about 60%) reported in the past [56,57].

#### 3.4.2. Air Permeability

The flow of air through a knitted fabric is described as air permeability. Most colorants did not affect the air permeability of the knitted fabric, but the blue 15:1 colorant was an exception (Table 7). The blue 15:1 colorant reduced the air permeability slightly, although the change was not significant. The observed change could also reflect the use of laboratory-scale knitting equipment, which may introduce small irregularities. Our results differ from previous reports involving exhaust dyeing, wherein surface modifications that improve the incorporation of dyes in knitted fabrics can affects the air permeability of fabrics more significantly because the dyes form covalent bonds with the polymer chains [18,58]. Colorant molecules interact with the polymer via a different mechanism during dope dyeing, so we did not observe a similar effect. Dope dying therefore offers more scope to control the comfort properties of fabrics, including air permeability.

#### 3.4.3. Martindale Abrasion Test

**Figure 12:**
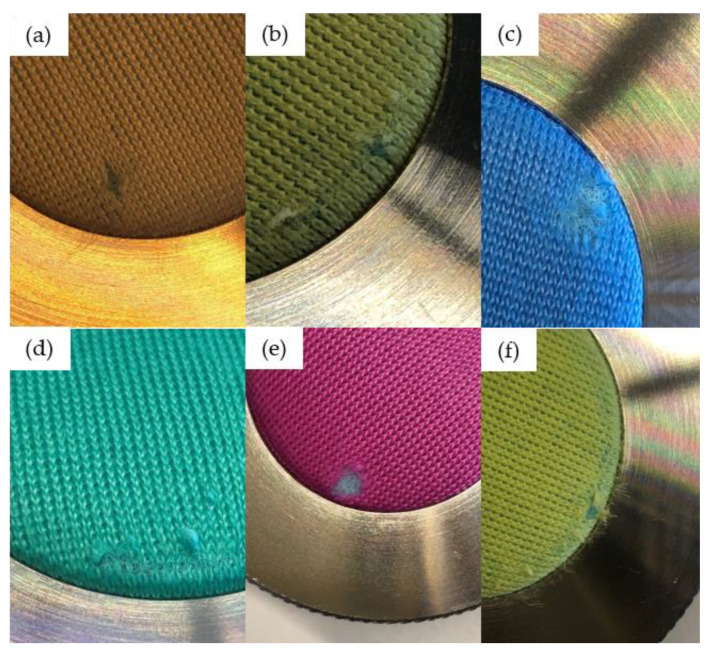
First signs of abrasion in knitted PLA fabrics: (**a**) PLA.1, (**b**) A03.1, (**c**) B03.1, (**d**) G03.1, (**e**) P03.1, and (**f**) Y03.1.

The first signs of abrasion were observed after 5000 cycles in the pure PLA fabrics, whereas more cycles (up to 10,000) were required to abrade the dyed fabrics (Figure 12 and Table 8). As stated above, these differences may reflect irregularities in the knitted surface introduced by the laboratory-scale knitting equipment. A value between 5000 and 10,000 cycles suggests that the fabrics produced here are more suited for decorative use (e.g., in cushions) than general use, which would require the fabrics to withstand >20,000 cycles [59]. Fabrics made from PLA blends have been reported to have low abrasion resistance, getting destroyed after 2250 cycles, but the yarn developed in this study was observed to have better abrasion resistance [60]. This can be the case because the yarn produced here had better mechanical properties compared to the PLA blend yarn used in the previous study. The processing conditions should therefore be optimized further to improve the abrasion resistance.

It is observed from Table 8 that the color fastness of PLA and the fabrics containing blue, green, pink, and yellow pigment is good. There was minimal color change after the Martindale abrasion test. However, in the case of fabric containing alizarin, a significant color change was observed and the fabric change from yellow to a light yellow color. The overall L,a,b values obtained herein are a little different from the values obtained from the yarn values. This could be the case because of the difference in the measurement method. In the case of yarn, the yarn was bundled into a ball, and the color was measured, and in the case of fabric, the color was measured directly from the reflection of light. This is hypothesized to lead to the small difference in color observed.

## 4. Conclusions

We investigated the effect of colorants on the structure and properties of dope-dyed PLA yarn and fabric. We produced master batches of PLA containing 5% (*w*/*w*) of each colorant using a twin-screw compounder and generated PLA yarns containing up to 0.3% (*w*/*w*) of the colorants using a pilot-scale melt spinning machine. This is a more efficient and less environmentally harmful approach to dye thermolabile biopolymers such as PLA. During spinning, we kept the winding speed constant but varied the melt composition and SSD ratio to investigate the effect of these parameters on the properties of PLA yarns. First, we analyzed the thermal properties and morphology of the colorants and found that no thermal transition occurred below the spinning temperature of 200 °C. We then analyzed the master batches to investigate the effect of colorants on the rheological and thermal properties of PLA, including its degradation. We observed that the presence of 5% (*w*/*w*) of the colorants led to an increase in viscosity. Furthermore, alizarin and the blue 15:1 and yellow 155 pigments showed nucleating activity. No change in molecular weight was observed in the master batches, suggesting the absence of PLA degradation. FTIR spectra revealed the possibility of hydrogen bonding between PLA and the colorants, which facilitated their distribution in the PLA matrix, as confirmed by SEM.

The mechanical testing of pure PLA yarns and yarns containing up to 0.3% (*w*/*w*) of the colorants spun at different drawing speeds revealed that the mechanical properties were affected most by the drawing speed, while the colorant had a negligible effect. Increasing the SSD ratio from 1 to 3 at a constant winding speed caused the tenacity of the yarn to increase by ~50%. DSC also revealed that drawing had a stronger influence on the degree of crystallinity of the yarns, but the colorant had no effect even if it acted as a nucleating agent. This may reflect the slow cooling rate during DSC (10 °C/min) compared to >200 °C/s during melt spinning, which is too rapid for nucleation to affect crystallization. Interestingly, the T_cc_ declined with increasing SSD ratio, suggesting that crystallization accelerates when there is already inherent orientation present in the processed yarn.

The BWS test revealed that all five colorants are UV-resistant. Furthermore, the incorporation of colorants had no effect on air permeability or water vapor permeability. The Martindale abrasion test revealed that the fabrics were suitable for decorative use. In conventional exhaust dyeing, the dyeing temperature and duration influence the mechanical properties, crystallinity, and the other physical aspects of knitted fabrics. In contrast, dope dyeing had little impact, allowing a greater degree of control over the yarn and fabric properties by modifying the process parameters. We also found that the potentially biobased colorant alizarin and the biobased pink PR122 pigment perform as well as the commercial colorants. We conclude that dope dyeing with colorants can be a sustainable alternative to conventional exhaust dyeing and offers better control of yarn properties.

## Figures and Tables

**Table 1:** Chemical structure of the colorants [23,24,25,26,27].

Additive	Chemical Structure
Alizarin	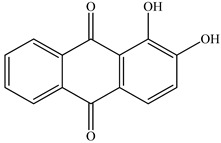
Pigment blue 15:1	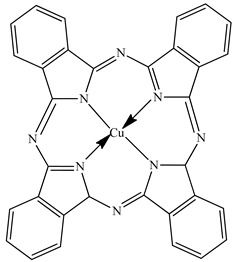
Pigment green 7	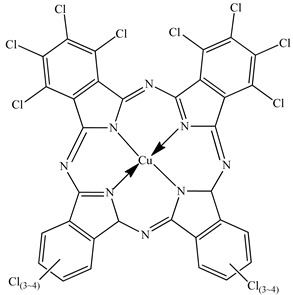
Pink PR122	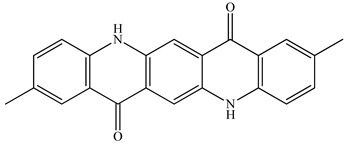
Pigment yellow 155	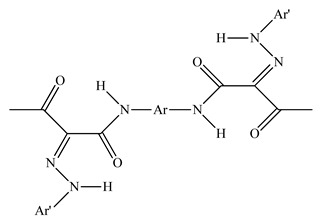

**Table 2:** Complete set of yarns produced under different experimental parameters.

Abbreviation	Material: Colorants Shown as % (*w*/*w*)	Take-Up (m/min)	Melt-Drawing Ratio	Winding Speed (m/min)	SSD Ratio
PLA.1	PLA	1100	72	1200	1
PLA.2	PLA	600	39	1200	2
PLA.3	PLA	400	26	1200	3
A03.1	PLA + 0.3% alizarin	1100	72	1200	1
A03.2	PLA + 0.3% alizarin	600	39	1200	2
A03.3	PLA + 0.3% alizarin	400	26	1200	3
B03.1	PLA + 0.3% blue 15:1	1100	72	1200	1
B03.2	PLA + 0.3% blue 15:1	600	39	1200	2
B03.3	PLA + 0.3% blue 15:1	400	26	1200	3
G01.1	PLA + 0.3% green 7	1100	72	1200	1
G02.2	PLA + 0.3% green 7	600	39	1200	2
G03.3	PLA + 0.3% green 7	400	26	1200	3
P03.1	PLA + 0.3% pink PR122	1100	72	1200	1
P03.2	PLA + 0.3% pink PR122	600	39	1200	2
P03.3	PLA + 0.3% pink PR122	400	26	1200	3
Y03.1	PLA + 0.3% yellow 155	1100	72	1200	1
Y03.2	PLA + 0.3% yellow 155	600	39	1200	2
Y03.3	PLA + 0.3% yellow 155	400	26	1200	3

**Table 3:** Tenacity and elongation of PLA and dyed yarns containing 0.3% (*w*/*w*) of each colorant with standard deviations (SD).

Material	Tenacity (cN/tex)	Elongation at Maximum Force (%)
PLA.1	13.90 ± 0.57	94.33 ± 5.08
PLA.2	17.53 ± 0.32	36.33 ± 1.75
PLA.3	20.03 ± 0.17	26.33 ± 1.22
A03.1	13.20 ± 0.08	95.67 ± 2.50
A03.2	17.60 ± 0.20	39.33 ± 1.74
A03.3	19.97 ± 0.41	31.00 ± 1.63
B03.1	11.97 ± 0.37	97.33 ± 1.14
B03.2	17.03 ± 0.17	40.33 ± 1.12
B03.3	19.60 ± 0.51	27.33 ± 1.72
G01.1	13.07 ± 0.18	100.00 ± 4.23
G02.2	16.17 ± 0.40	39.67 ± 2.34
G03.3	19.33 ± 0.24	29.33 ± 0.98
P03.1	14.10 ± 0.37	100.00 ± 1.09
P03.2	16.63 ± 0.72	32.33 ± 0.42
P03.3	19.67 ± 1.18	25.00 ± 2.13
Y03.1	11.77 ± 0.16	95.00 ± 0.94
Y03.2	16.27 ± 0.34	34.00 ± 3.11
Y03.3	18.30 ± 0.38	25.33 ± 2.25

**Table 4:** The thermal transition values of PLA and dyed yarns drawn at different SSD ratios with SD (*n* = 3).

Sample	T_g_ (°C)	T_cc_ (°C)	T_m_ (°C)	X_c_ (%)	T_c_ (°C)
PLA.1	60.8 ± 0.2	79.5 ± 0.5	174.2 ± 0.9	29.8 ± 2.1	100.2 ± 0.5
PLA.2	60.5 ± 0.5	79.3 ± 0.6	174.3 ± 0.6	29.9 ± 1.3	100.2 ± 1.3
PLA.3	-	75.0 ± 0.1	173.0 ± 1.0	51.7 ± 3.1	100.2 ± 0.9
A03.1	60.1 ± 0.3	82.7 ± 1.2	175.2 ± 0.6	27.6 ± 3.0	101.0 ± 0.7
A03.2	60.5 ± 0.1	81.3 ± 1.7	174.9 ± 1.0	29.7 ± 1.7	97.7 ± 1.5
A03.3	-	75.8 ± 0.4	176.0 ± 1.5	51.5 ± 5.3	99.1 ± 1.0
B03.1	60.2 ± 1.5	84.3 ± 0.8	174.4 ± 0.3	30.7 ± 0.8	133.1 ± 0.4
B03.2	61.2 ± 0.7	80.6 ± 2.0	173.9 ± 0.7	27.1 ± 1.5	133.8 ± 0.6
B03.3	-	74.8 ± 0.3	172.8 ± 0.4	48.5 ± 3.1	133.4 ± 0.8
G03.1	59.8 ± 1.8	82.5 ± 0.2	173.8 ± 0.4	29.3 ± 0.9	104.7 ± 0.2
G03.2	60.2 ± 1.9	79.8 ± 1.3	174.6 ± 0.6	30.4 ± 0.9	105.2 ± 0.2
G03.3	-	74.7 ± 0.3	173.3 ± 0.7	44.9 ± 1.2	104.0 ± 0.2
P03.1	60.0 ± 0.5	79.9 ± 0.7	174.6 ± 0.1	29.9 ± 1.9	108.7 ± 1.4
P03.2	61.1 ± 0.2	78.4 ± 1.1	176.0 ± 0.8	29.1 ± 0.9	108.0 ± 0.9
P03.3	-	-	173.7 ± 0.2	52.1 ± 0.1	106.2 ± 0.6
Y03.1	60.2 ± 3.2	82.8 ± 0.5	174.6 ± 0.1	29.7 ± 0.3	127.2 ± 1.0
Y03.2	61.8 ± 0.3	79.9 ± 1.5	174.3 ± 0.8	30.0 ± 1.3	127.5 ± 0.9
Y03.3	-	74.8 ± 0.5	173.1 ± 0.2	48.47 ± 2.1	127.7 ± 1.0

**Table 5:** CIE L*a*b color values and blue wool scale (BWS) of PLA and dyed yarns.

Material	L	a	b	BWS
PLA-3	84.25	–0.42	2.23	≥7
A01-3	84.46	–2.8	22.19	-
A02-3	83.71	–4.12	41.78	-
A03-3	80.13	–2.67	53.66	≥7
B01-3	41.30	7.53	–31.81	-
B02-3	47.00	8.18	–32.21	-
B03-3	37.09	3.31	–25.22	≥7
G01-3	80.13	–25.83	1.66	-
G02-3	73.19	–43.85	3.44	-
G03-3	71.64	–51.32	3.62	≥7
P01-3	69.17	29.29	–14.61	-
P02-3	60.85	41.12	–19.52	-
P03-3	54.07	49.1	–22.34	≥7
Y01-3	85.06	–5.62	41.44	-
Y02-3	84.37	–4.94	30.93	-
Y03-3	85.77	–5.45	54.75	≥7

**Table 6:** Relative and absolute water vapor permeability of knitted fabrics made from PLA and dyed PLA yarns containing 0.3% (*w*/*w*) of colorants. Data are means ± SD (*n* = 5).

Material	Relative Water Vapor Permeability (%)	Absolute Water Vapor Permeability (Pa/m^2^/W^−1^)
PLA	71.34 ± 3.04	2.60 ± 0.32
A03.1	70.30 ± 1.54	2.46 ± 0.19
B03.1	71.76 ± 3.16	2.72 ± 0.46
G03.1	73.56 ± 2.85	2.46 ± 0.35
P03.1	75.36 ± 2081	2.22 ± 0.32
Y03.1	74.60 ± 2.60	2.38 ± 0.31

**Table 7:** Absolute air permeability of knitted fabrics made from PLA and dyed PLA yarns containing 0.3% (*w*/*w*) of each colorant. Data are means ± SD (*n* = 5).

Material	Air Permeability (L/m^2^/s)
PLA	3848 ± 332
A03.1	3406 ± 65
B03.1	3026 ± 208
G03.1	3890 ± 116
P03.1	3506 ± 257
Y03.1	3906 ± 297

**Table 8:** Abrasion resistance and color fastness of PLA and dyed fabrics containing 0.3% (*w*/*w*) of each colorant, determined using the Martindale abrasion test.

Material	Abrasion Resistance (Number of Cycles)	Before Martindale			After Martindale		
		L	a	b	L	a	b
PLA	5000	78.50	−0.21	1.02	72.66	−1.47	3.67
A03.1	10,000	76.80	−2.15	45.20	63.36	−1.00	25.51
B03.1	7500	31.09	3.22	26.22	30.22	5.13	28.66
G03.1	10,000	65.22	−38.22	2.12	63.11	−35.56	2.25
P03.1	7500	44.94	50.05	−13.27	42.91	38.06	−13.23
Y03.1	7500	73.97	−11.13	40.77	70.23	−9.48	33.64

## Data Availability

The datasets used and/or analyzed during this study are available from the corresponding author on reasonable request.
